# ﻿Two new freshwater species of the genus *Achnanthidium* (Bacillariophyta, Achnanthidiaceae) from Qingxi River, China

**DOI:** 10.3897/phytokeys.191.78489

**Published:** 2022-03-04

**Authors:** Pan Yu, Qingmin You, Wanting Pang, Quanxi Wang

**Affiliations:** 1 College of Life and Environmental Sciences, Shanghai Normal University, Shanghai 200234, China Shanghai Normal University Shanghai China; 2 Institute of Hydrobiology, Chinese Academy of Sciences, Wuhan, 430072, China Institute of Hydrobiology, Chinese Academy of Sciences Wuhan China

**Keywords:** Diatoms, monoraphid, morphology, new species, taxonomy

## Abstract

We describe two new *Achnanthidium* species, *A.anhuense***sp. nov.** and *A.qingxiense***sp. nov.**, from Qingxi River, Anhui Province, China, based on light and scanning electron microscopy. Both species belong to the “*A.pyrenaicum* complex” of the *Achnanthidium* genus, based on their possession of transapically-elongated areolae and deflected external distal raphe fissures. *A.anhuense***sp. nov.** has a slightly irregular linear-lanceolate valve with rounded or weakly protracted apices and a transapically rectangular or bow tie central area on the raphe valve. *A.qingxiense***sp. nov.** has a linear-lanceolate valve with rounded apices and the axial area distinct expanded apices on the rapheless valve. Both species differ sufficiently from other similar species, based on valve outline, shape of the axial and central areas and striae density. These new species were all collected from stone substratum.

## ﻿Introduction

The diatom genus *Achnanthidium*Kützing (1844) was originally described by Kützing, as a subgenus of the *Achnanthes*Bory de Saint-Vincent (1822). The species *Achnanthesmicrocephalum* Kützing was the type species of the subgenus (Pérès et al. 2014; Yu et al. 2019a). In the 1990s, Round et al. (1990) resurrected *Achnanthidium* and elevated it to the genus level. Later, Round and Bukhtiyarova (1996) redefined the circumscription of this genus, with the main identification characteristics of *Achnanthidium* including small cells with a length and width of usually less than 30 µm and 5 µm, respectively, linear-lanceolate to lanceolate-elliptic valves and straight or curved external distal raphe ends (Round and Bukhtiyarova 1996; Yu et al. 2019 a, b). Species of *Achnanthidium* are widely distributed in various types of freshwater habitats in which they are common and abundant (Novais et al. 2011; Pinseel et al. 2015; Karthick et al. 2017; You et al. 2019; Yu et al. 2019a, b).

Owing to the small size (usually less than 30 µm in length and less than 5 µm in breadth) and identification characteristics of *Achnanthidium*, this genus has been subdivided into three major subgroups. The *A.minutissimum* complex includes species with straight external distal raphe ends and linear to linear-lanceolate valve shapes, increasing striae density toward the apices and round external areolar openings. The species of the *A.pyrenaicum* complex have external distal raphe ends that are deflected or hooked to one side of the valve and slit-like areolar openings. The members of the *A.exiguum* complex have external distal raphe ends curved in opposite directions (Compère and Van de Vijver 2011; Karthick et al. 2017; Yu et al. 2018, 2019a; Miao et al. 2020; Tseplik et al. 2021; You et al. 2021). *A.exiguum* and its relatives have been segregated into a new genus of *Gogorevia* Kulikovskiy, Glushchenko, Maltsev and Kociolek (Kulikovskiy et al. 2020).

Presently, the number of species in the genus *Achnanthidium* is greater than 200 (Marquardt et al. 2017; Kociolek et al. 2018; You et al. 2021). Before the year of 2000, 11 new *Achnanthes* species had been described from China (Hustedt 1922; Jao 1964; Jao et al. 1974; Qi and Xie 1984; Zhu and Chen 1994, 1996; Liu et al. 2021). Some of these species should be transferred to *Achnanthidium*, but owing to the loss of the type material, it is difficult to confirm their taxonomic position. It is, therefore, necessary to collect samples from the type locality, and re-evaluate their taxonomic position (Liu et al. 2021). From 2001 to 2021, 14 new *Achnanthidium* species have been described from China (Liu et al. 2016; Yu et al. 2018, 2019a, b, You et al. 2019, 2021; Liu et al. 2021). During an investigation of the freshwater diatoms from the Qingxi River, two unknown *Achnanthidium* species were discovered. The purpose of this present study was to document and formally describe those species with light microscopy (LM) and scanning electron microscopy (SEM) and compare the new species with morphologically similar taxa.

## ﻿Materials and methods

Diatom samples were collected from the Qingxi River located in Chizhou City, Anhui Province, China, in January 2018. In the field, several water chemistry parameters were recorded, including pH, temperature, dissolved oxygen, salinity, total dissolved solids (TDS) and conductivity, using a YSIPro Plus multiparameter meter (YSI, Ohio, USA). Diatom samples were collected from stones using clean toothbrushes and the samples were placed in a bottle and preserved with formalin (4% final concentration). Total phosphorus (TP) was measured by alkaline potassium persulphate digestion ultraviolet spectrophotometry, total nitrogen (TN) was measured by potassium persulphate digestion ammonium molybdate spectrophotometry and chemical oxygen demand (COD) was measured with the potassium permanganate index method (CSEPB 2002).

In the laboratory, diatom samples were cleaned with concentrated nitric acid using the Microwave Accelerated Reaction System (Model MARS, CEM Corporation, Charlotte, USA) (Parr et al. 2004) with a pre-programmed digestion scheme (temperature, 180 °C) (Yu et al. 2017, 2019a, b). Next, samples were alternately centrifuged for 5 min at 3500 rpm (TDZ5-WS, Luyi Corporation, Shanghai, China) and washed six times using distilled water until the pH of the sample was close to neutral. Finally, the cleaned samples were kept in 95% ethanol. Cleaned diatom frustules were mounted in Naphrax for LM or air-dried on to cover slips and mounted on to alloy stubs for observation by SEM. The LM observations were made with an Olympus BX-53 microscope (Tokyo, Japan) fitted with DIC optics and a 100× oil immersion objective (1.4 numerical aperture) and an Olympus DP-71 digital camera. The SEM examination was conducted using a Hitachi SU8010 (1–2 kV, WD 8 mm) (Tokyo, Japan). Images were compiled with Adobe Photoshop CS6. Morphological terminology followed Round et al. (1990). All of the diatom samples and permanent slides are housed in the Lab of Algae and Environment, College of Life Sciences, Shanghai Normal University (SHTU), Shanghai, China.

## ﻿Results

### ﻿ Achnanthidium anhuense

Taxon classificationPlantaeCocconeidalesAchnanthidiaceae

P. Yu, Q. M. You & Q. X. Wang sp. nov.

713455CD-87A3-5BE2-92DF-7AE03D3F88DC

Figs 1A –AD , 2 –5

#### Description.

**Figures 1.**
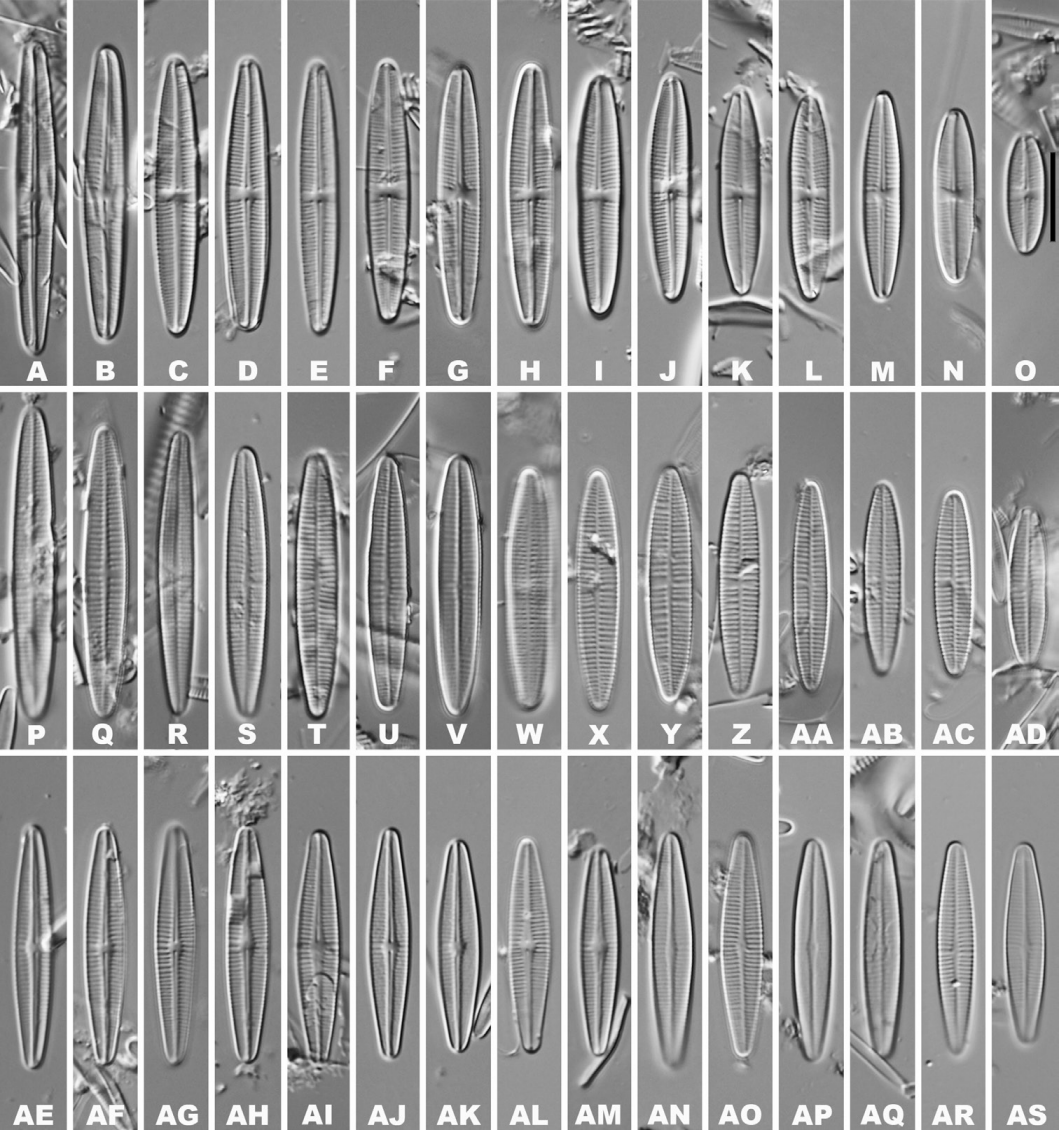
**A–AD**LM valve views of *Achnanthidiumanhuense* sp. nov. **AE–AS**LM valve views of *Achnanthidiumqingxiense* sp. nov. Scale bar: 10 µm.

**LM** (Fig. 1A–AD), valves are slightly irregularly linear-lanceolate in shape, with rounded or weakly protracted apices. Valve length 13–35.7 µm, breadth 3.5–4.5 µm (n = 200). Raphe valve is concave, with narrow, linear-lanceolate axial area, with a central area that is transapically rectangular or bow tie-shaped, usually slightly asymmetric. Striae radiate at the middle portion and nearly parallel towards apices, the number of striae is 18–20 in 10 µm at the middle portion, 26–32 in 10 µm near the apices. Rapheless valve is convex, axial area narrow linear-lanceolate and weakly expanded at the middle portion of the valve. Striae are nearly parallel, 16–26 in 10 µm in the centre and 22–30 in 10 µm near the apices.

**Figures 2.**
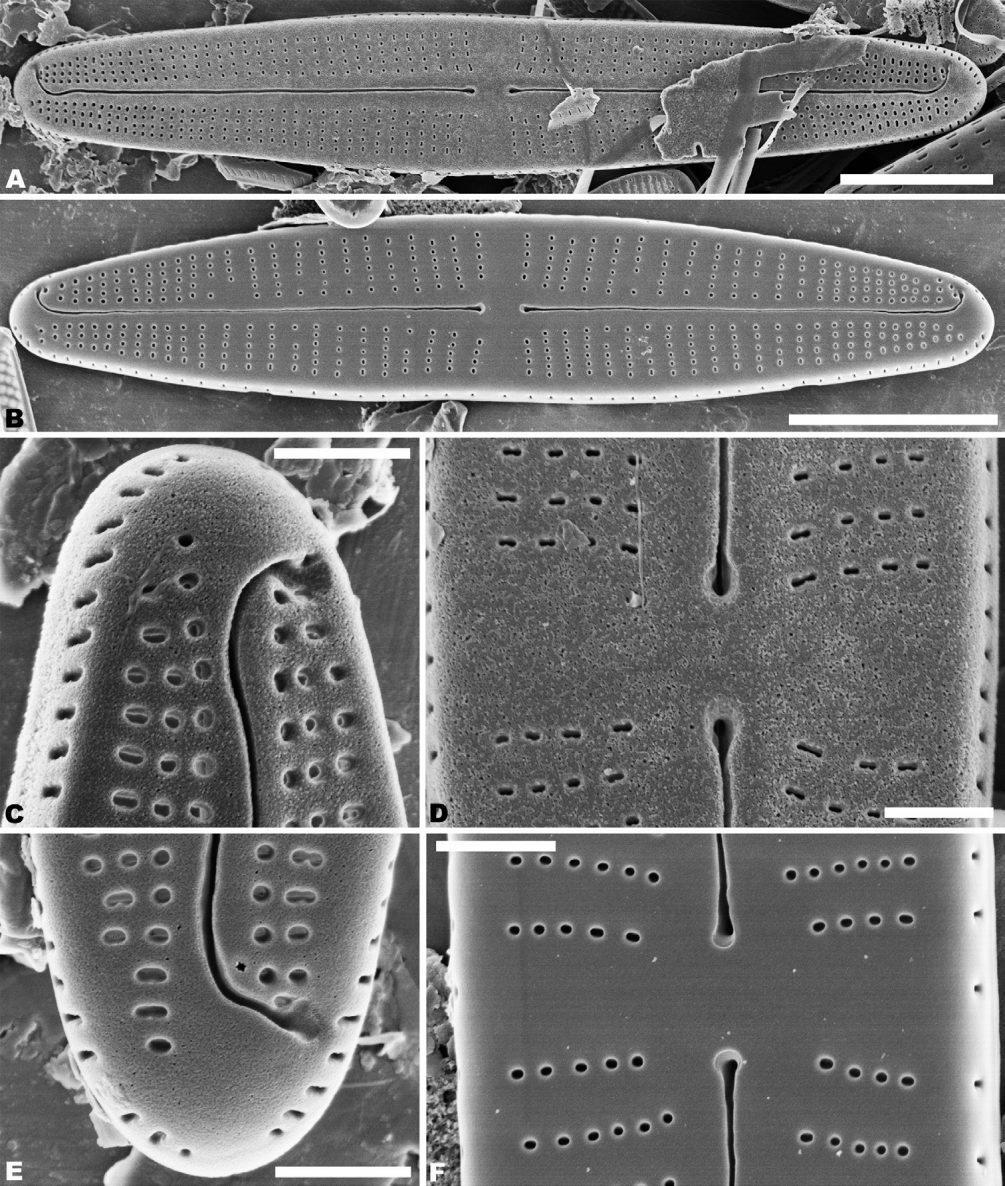
**A–F***Achnanthidiumanhuense* sp. nov., SEM external views of raphe valve **A, B** external view of an entire raphe valve **C, E** apices of the valve, showing the distal raphe ends **D, F** central area of the valve, showing the proximal raphe ends, **D** seems detail of **A** and **F** of **B**. Scale bars: 5 µm (**A, B**); 1 µm (**C–F**).

**Figures 3.**
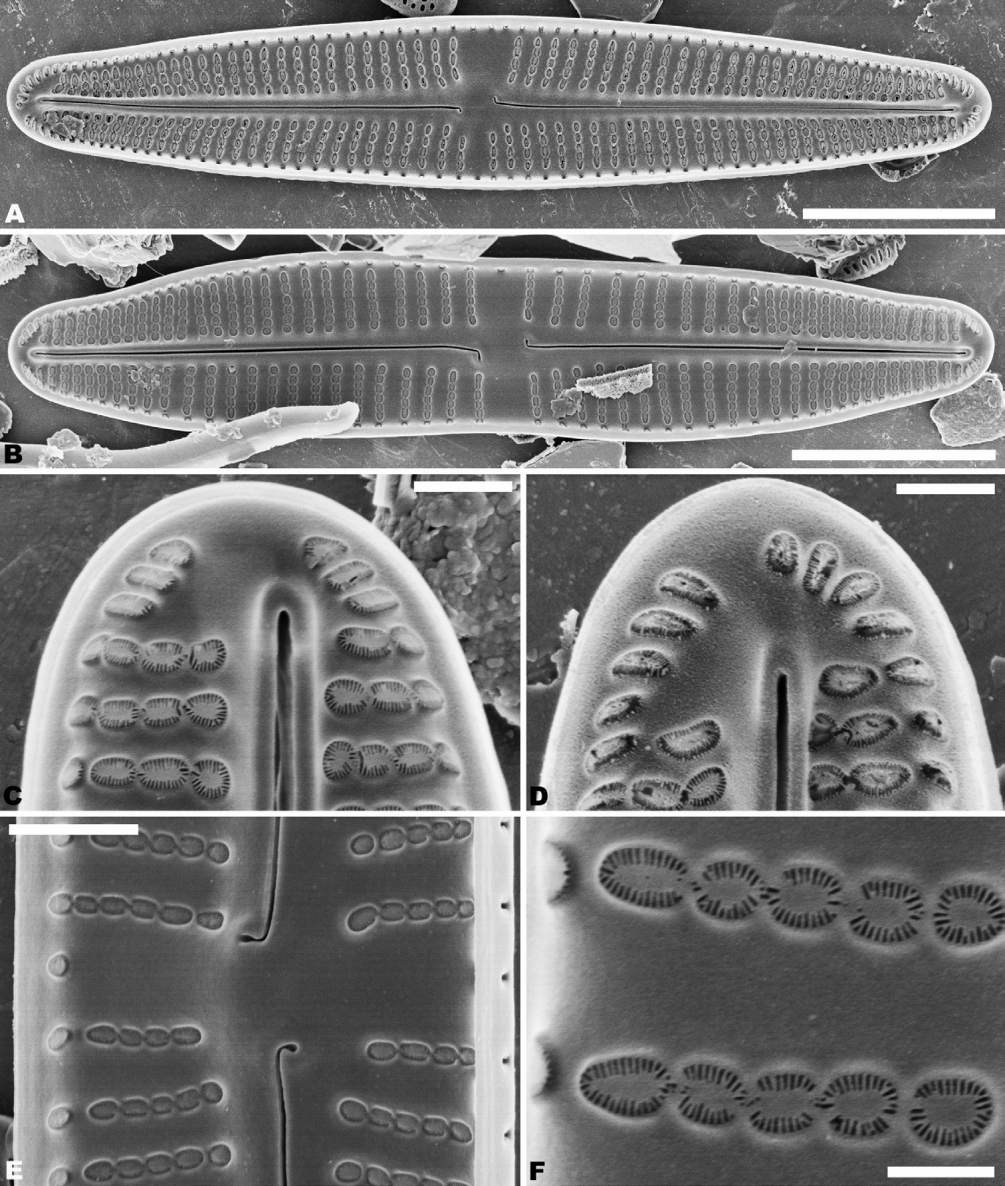
**A–F***Achnanthidiumanhuense* sp. nov., SEM internal views of raphe valve **A, B** internal view of an entire raphe valve **C, D** apices of the valve, showing the distal raphe ends **E** central area of the valve, showing the proximal raphe ends **F** internal areola openings with fine hymenate structures. Scale bars: 5 µm (**A, B**); 1 µm (**E**); 0.5 µm (**C, D**); 0.3 µm (**F**).

**Figures 4.**
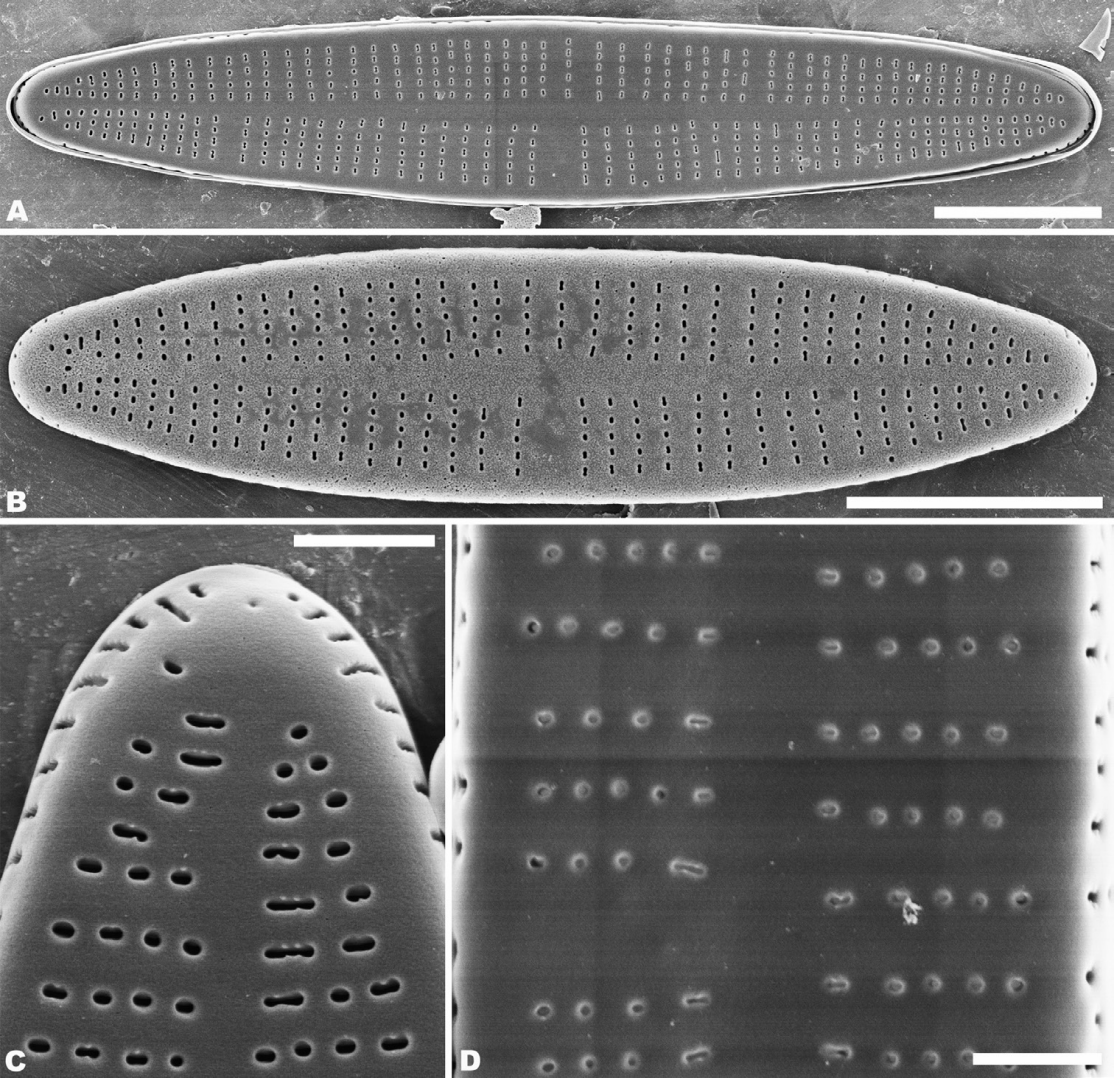
**A–D***Achnanthidiumanhuense* sp. nov., SEM external views of rapheless valve **A, B** external view of an entire rapheless valve **C** apices of the valve **D** central area of the valve. Scale bars: 5 µm (**A, B**); 1 µm (**C, D**).

In the SEM, both valves have a narrow hyaline area at the valve face and mantle junction (Figs 2A, B, 4A, B). Raphe valve: Externally, the raphe is filiform and straight (Fig. 2A, B), distal raphe ends are deflected to the same side (Fig. 2A–C, E), and proximal raphe ends straight and teardrop-shaped (Fig. 2A, B, D, F). Striae uniseriate, containing 3–6 round, oval or transapically-orientated areolae in the middle portion of the valve and 1–3 round, oval or transapically-orientated areolae at the apices (Fig. 2A–C, E). Valve mantle with a single row of linear areolae extend along the valve, but with a slight interruption in the apices (Fig. 2B, C, E). Internally, raphe terminates in raised helictoglossae close to the apices (Fig. 3A–D), proximal raphe ends form small hooks and are distinctly deflected in opposite directions (Fig. 3A, B, E). Areolae transapically elongated in the central portion of the valve, becoming larger and oblong at the apices (Fig. 3C–E). Areolae are occluded by hymenes perforated by delicate slits and each hymene joins with the adjacent hymene (Fig. 3F).

**Figures 5.**
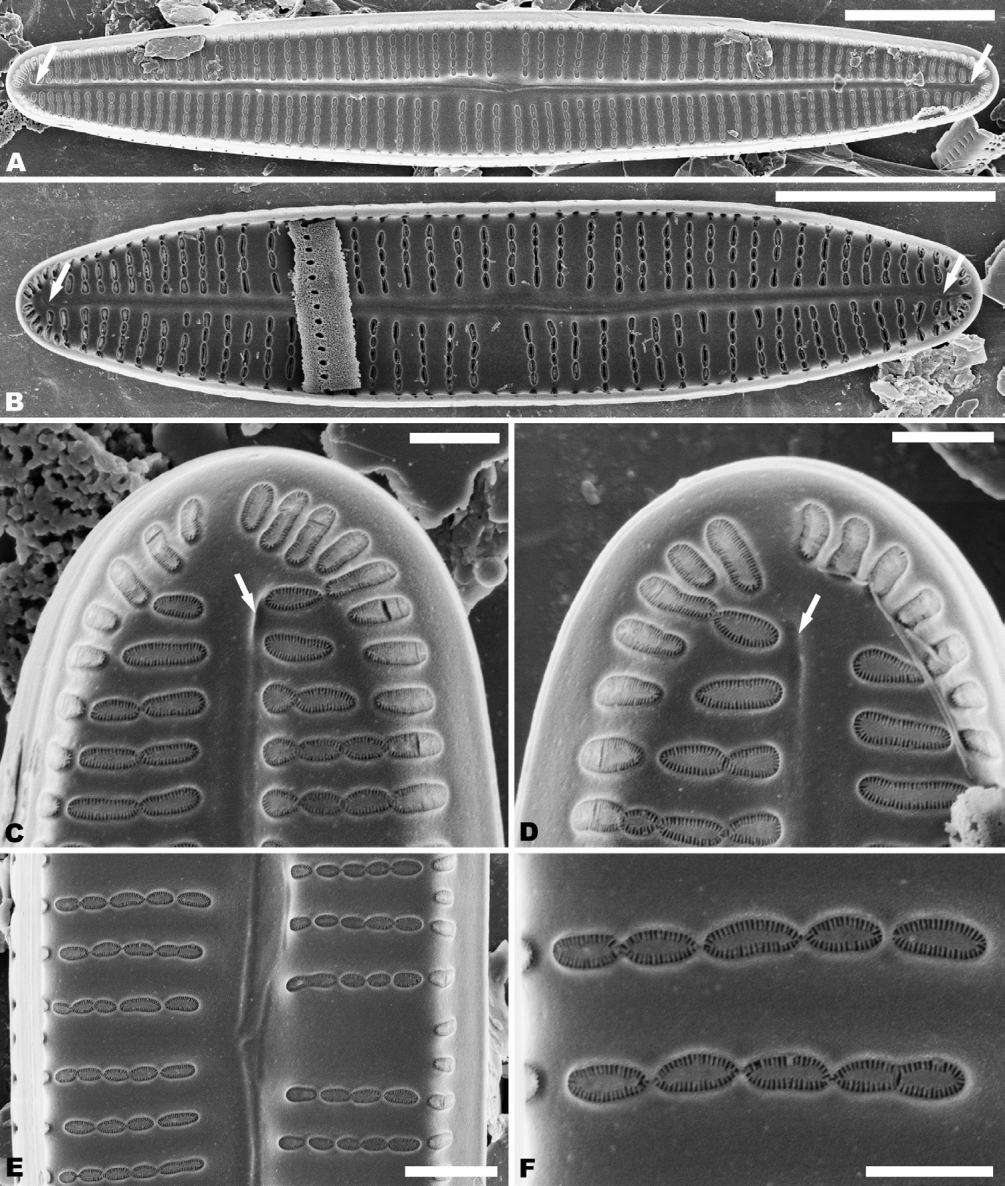
**A–F***Achnanthidiumanhuense* sp. nov., SEM internal views of rapheless valve **A, B** internal view of an entire rapheless valve **C, D** apices of the valve **E** central area of the valve **F** internal areola openings with fine hymenate structures. Scale bars: 5 µm (**A, B**); 1 µm (**E**); 0.5 µm (**C, D, F**).

Rapheless valve: Externally, the axial area is linear, being weakly expanded in the central area (Fig. 4A, B). Striae are uniseriate, comprise of 4–6 round or transapically elongated areolae in the central area (Fig. 4A, B, D) and 1–4 round or irregular oblong areolae at the apices (Fig. 4A–C). A row of slit-like areolae is present on the mantle (Fig. 4C, D). Internally, axial area slightly raised, with a shallow cutting line at the middle portion (Fig. 5A, B, E) and have a shallow depression at the ends of the axial area (Fig. 5A–D). Areolae are transapically oval in the centre of the valve (Fig. 5A, B, E) and large, irregular and oblong at the ends (Fig. 5A–D). Areolae are occluded by hymenes perforated by delicate slits and each hymene joins with the adjacent hymene (Fig. 5F).

#### Holotype

**(designated here).**SHTU! Slide QXH201801-Z7 in Lab of Algae and Environment, College of Life Sciences, Shanghai Normal University, Shanghai, China. Holotype illustrated in Fig. 1H, W.

#### Registration.

http://phycobank.org/103057

#### Type locality.

China. Qingxi River, Anhui Province, 30°14'39"N, 117°49'58"E, *leg. Q.X. Wang and P. Yu*, *23^th^ January 2018*.

#### Etymology.

The species is named for the place where it was found, namely Anhui Province.

#### Ecology.

Collected in one sample (QXH201801-Z7) on stone. Water temperature – 8.1 °C, pH – 7.8, Salinity – 0.05‰, TDS – 95.55 mg·l^-1^, EC – 99.3 μS·cm^–1^, TN – 0.5 mg·l^-1^, TP – 0.03 mg·l^-1^, COD – 0.1 mg·l^-1^.

#### Distribution.

So far only known from the type locality.

### ﻿ Achnanthidium qingxiense

Taxon classificationPlantaeCocconeidalesAchnanthidiaceae

Q. M. You, P. Yu & Q. X. Wang sp. nov.

4341DFD2-EB4A-52C8-B84D-480F5AA60F23

Figs 1AE –AS , 6 –9

#### Description.

**LM** (Fig. 1AE–AS). Valves linear-lanceolate in shape, with rounded or weakly protracted apices. The valve length is 22.5–28 µm and breadth of 3.8–4.6 µm (n = 30). Raphe valve with a narrow, linear-lanceolate axial area is weakly expanded at the middle portion of the valve. Striae slightly radiate at the centre area, becoming denser towards the apices, 21–25 in 10 µm at the centre, 42–44 in 10 µm near the apices. Rapheless valve with narrow, linear axial area is weakly expanded at the middle portion of the valve. Striae are nearly parallel, becoming denser towards the apices, 20–24 in 10 µm at the centre, up to 32–34 in 10 µm at the apices.

**Figures 6.**
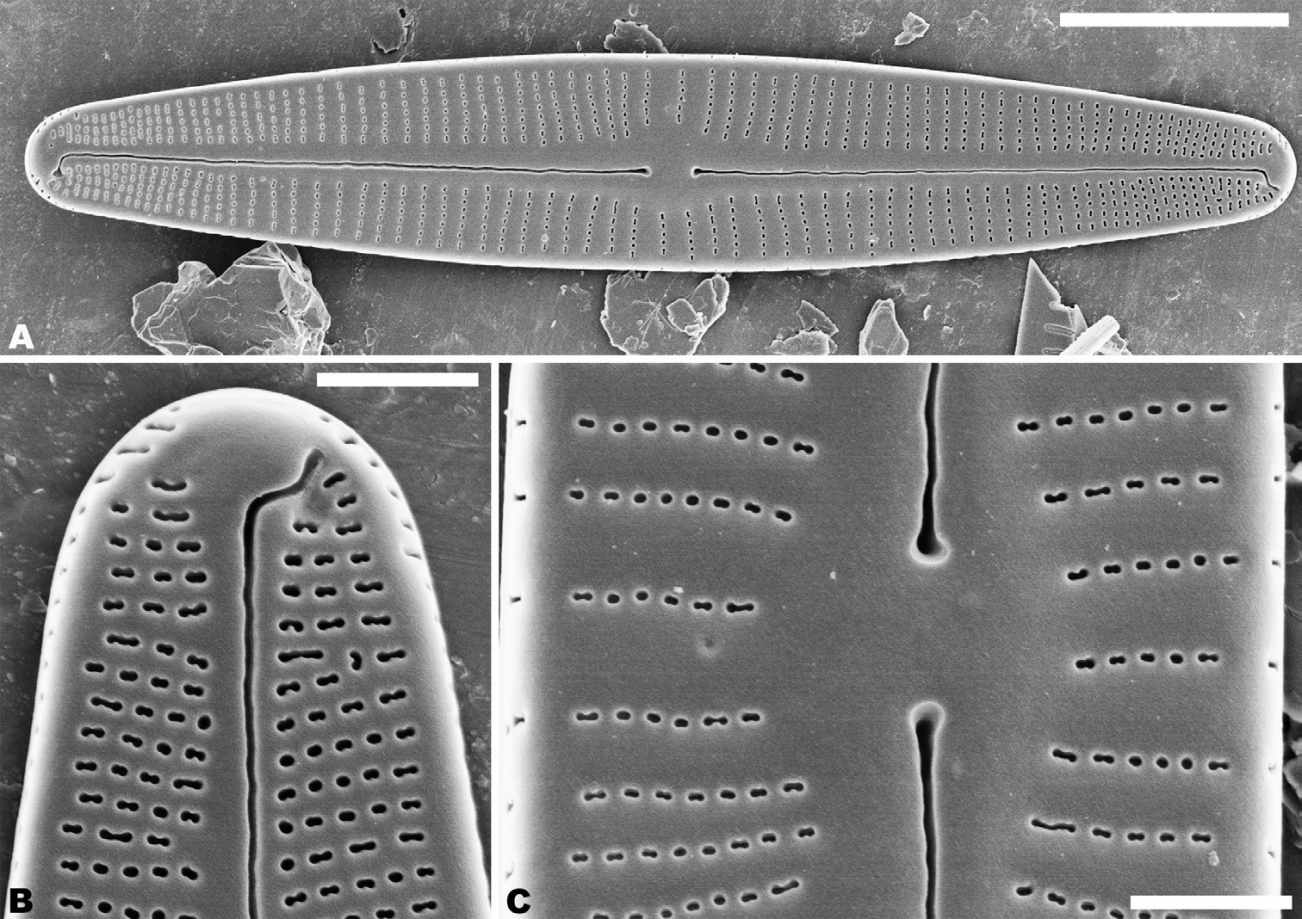
**A–C***Achnanthidiumqingxiense* sp. nov., SEM external views of raphe valve **A** external view of an entire raphe valve **B** apices of the valve, showing the distal raphe ends **C** central area of the valve, showing the proximal raphe ends. Scale bars: 5 µm (**A**); 1 µm (**B, C**).

**Figures 7.**
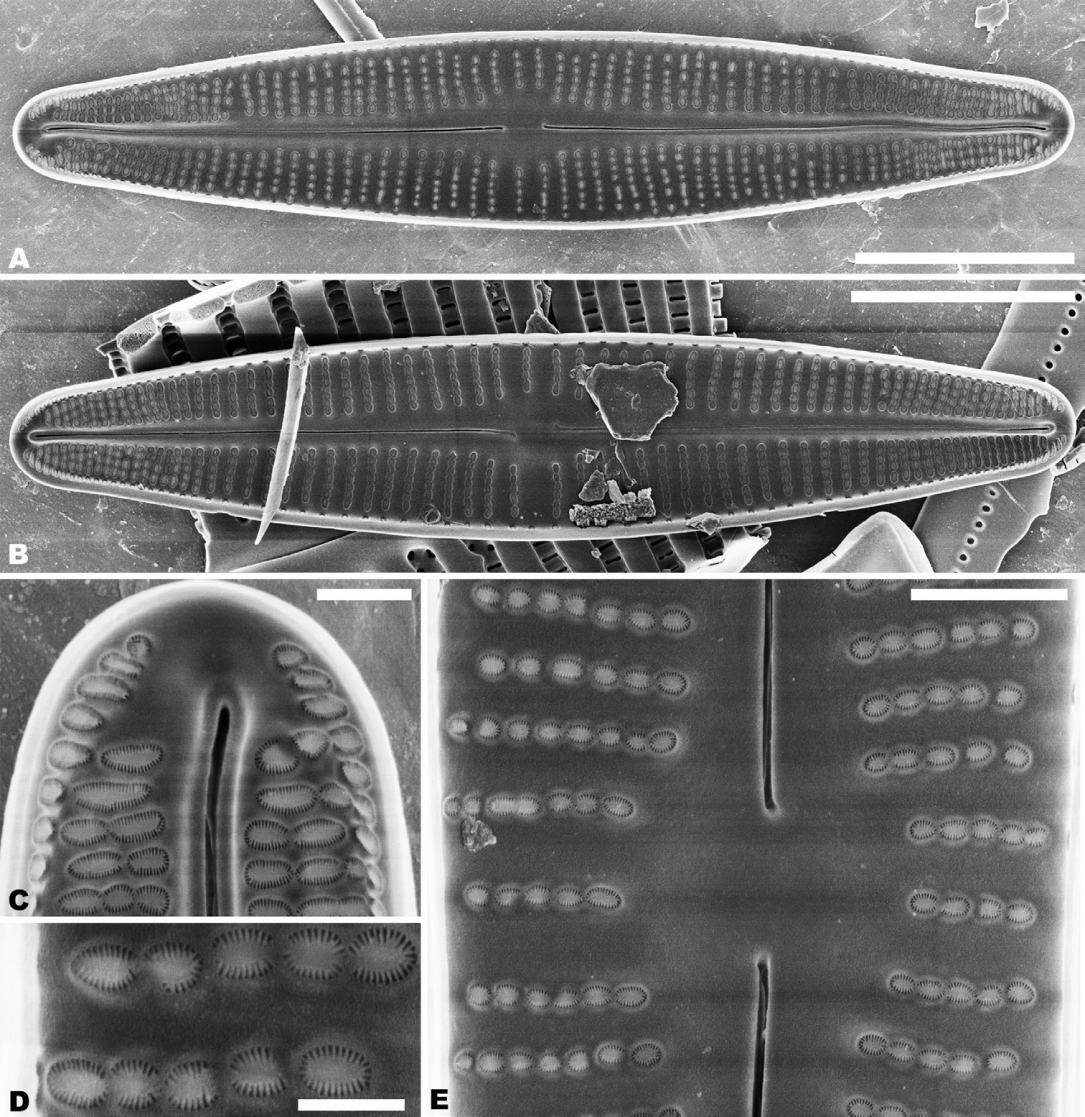
**A–E***Achnanthidiumqingxiense* sp. nov., SEM internal views of raphe valve **A, B** internal view of an entire raphe valve **C** apices of the valve, showing the distal raphe ends **E** central area of the valve, showing the proximal raphe ends **D** internal areola openings with fine hymenate structures. Scale bars 5 µm (**A, B**); 1 µm (**C, E**); 0.3 µm (**D**).

**Figures 8.**
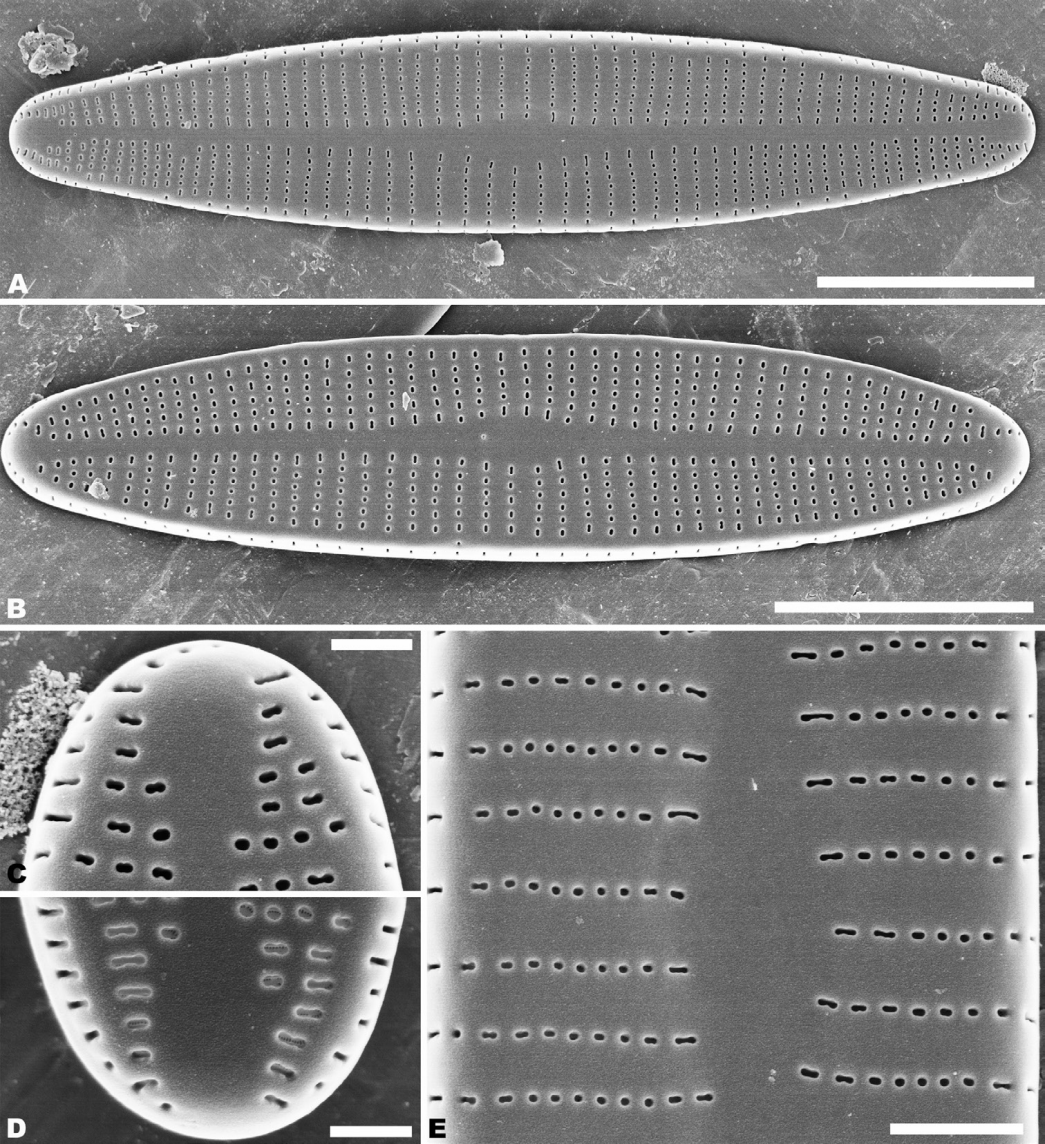
**A–E***Achnanthidiumqingxiense* sp. nov., SEM external views of rapheless valve **A, B** external view of an entire rapheless valve **C, D** apices of the valve **E** central area of the valve. Scale bars: 5 µm (**A, B**); 1 µm (**E**); 0.5 µm (**C, D**).

In the SEM, on both valves, the valve mantle has a single row of slit-like areolae (Figs 6B, C, 8A, B). Externally, the raphe is filiform, slightly undulate and has distal raphe ends deflected to the same side (Fig. 6A). On the side of the deflection, there is a depression near the distal raphe ends (Fig. 6A, B). The proximal raphe ends are straight and teardrop-shaped (Fig. 6A, C). Areolae are small, round to transapically orientated, the uniseriate striae are composed of 5–8 areolae in the middle portion of the valve (Fig. 6A, C) and 1–5 areolae at the apex (Fig. 6A, B). Internally, distal raphe ends terminate in raised helictoglossae (Fig. 7A–C), while the proximal raphe ends are weakly deflected in opposite directions (Fig. 7A, B, E). Areolae transapically elongated in the central portion of the valve and becoming larger and oblong at the apices (Fig. 7A–C, E).

**Figures 9.**
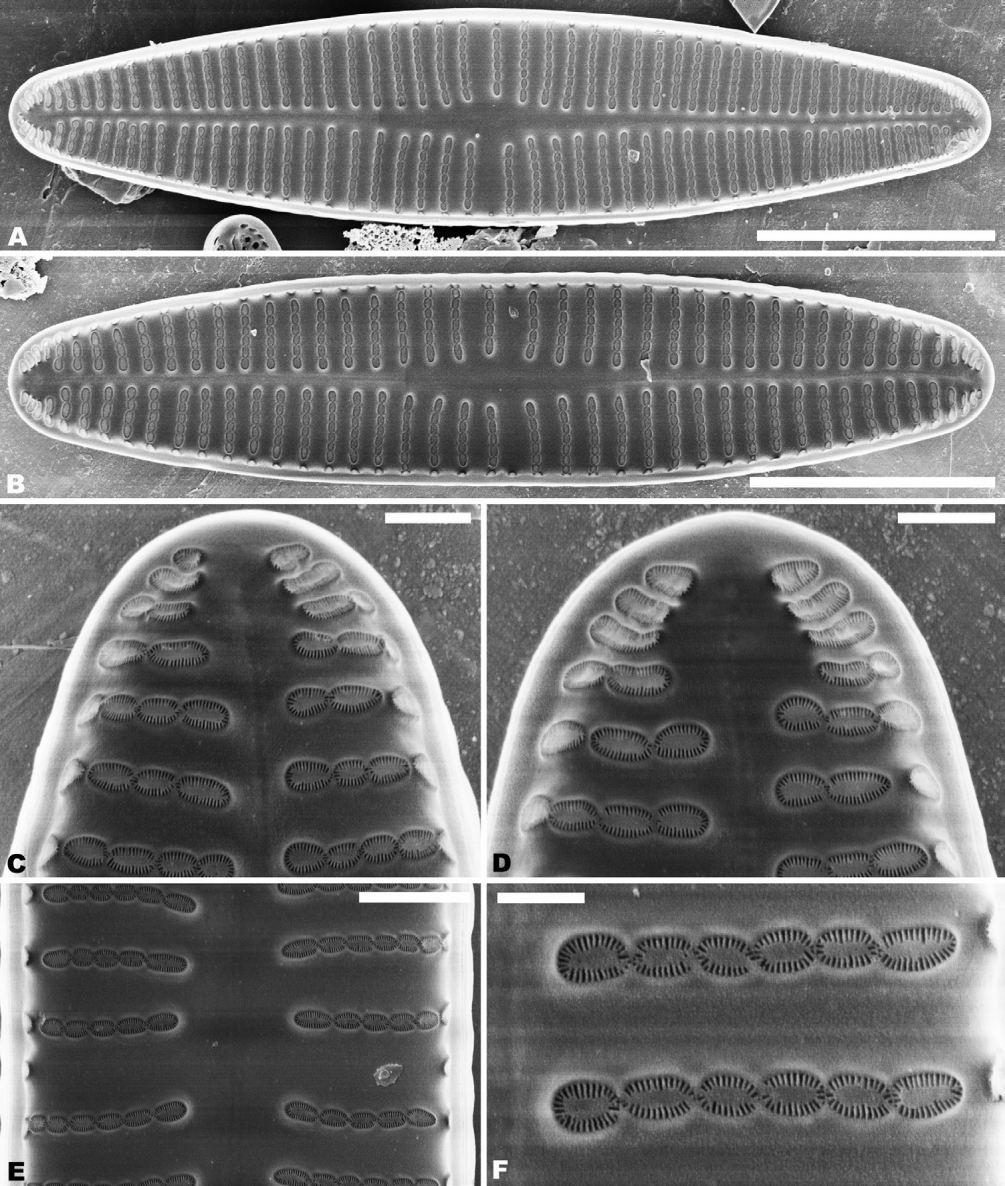
**A–F***Achnanthidiumqingxiense* sp. nov., SEM internal views of rapheless valve **A, B** internal view of an entire rapheless valve **C, D** apices of the valve **E** central area of the valve **F** internal areola openings with fine hymenate structures. Scale bars: 5 µm (**A, B**); 1 µm (**E**); 0.5 µm (**C, D**); 0.3 µm (**F**).

Rapheless valve: Externally, the axial area is linear, being weakly expanded at the central area and distinctly expanded at the apices (Fig. 8A–E). Striae are uniseriate, comprise of 5–10 round or transapically orientated areolae in the central area (Fig. 8A, B, E) and 1–4 round, oblong or slit-like areolae at the apices (Fig. 8A–D). Internally, the axial area is slightly raised, with a subtriangular area at the ends of the axial area (Fig. 9A–D). Areolae are transapically oval in the centre of the valve (Fig. 9A, B, E) and large, irregular and oblong at the ends (Fig. 9A–D). On both interiors of both valves, areolae are occluded by hymenes perforated by delicate slits and each hymene joins with the adjacent hymene (Figs 7D, 9F).

#### Holotype

**(designated here).**SHTU! Slide QXH201801-Z7 in Lab of Algae and Environment, College of Life Sciences, Shanghai Normal University, Shanghai, China. Holotype illustrated in Fig. 1AJ, AO.

#### Registration.

http://phycobank.org/103058

#### Type locality.

China. Qingxi River, Anhui Province, 30°14'39"N, 117°49'58"E, *leg. Q.X. Wang and P. Yu*, *23^th^ January 2018*.

#### Etymology.

The species is named after Qingxi River, where it was discovered.

#### Ecology.

Collected in one sample (QXH201801-Z7) on stone. The environmental conditions are exactly the same as for the *A.dubium* sp. nov.

#### Distribution.

The new species is known only from the type locality.

## ﻿Discussion

Based on the generic description of *Achnanthidium* by Round et al. (1990) and Round and Bukhtiyarova (1996), *A.anhuense* sp. nov. and *A.qingxiense* sp. nov. clearly belong to this genus. The two new species possess characters that support their assignment to the *A.pyrenaicum* complex, deflected external distal raphe fissures (Yu et al. 2018, 2019 b, You et al. 2019, 2021).

*Achnanthidiumanhuense* sp. nov. is similar to a few species, including *A.pyrenaicum* (Hustedt) Kobayasi (Kobayasi 1997), *A.pseudoconspicuum* (Foged) Jüttner & Cox (Jüttner, Cox 2011), *A.rostropyrenaicum* Jüttner & Cox (Jüttner et al. 2011) and *A.initium* Karthick, Taylor and Hamilton (Karthick et al. 2017). To facilitate a comparison between *A.anhuense* sp. nov. and these similar species, their morphological characteristics are summarised in Table 1. There is a difference in the valve outline between *A.anhuense* sp. nov. and these other species, with the valves of *A.anhuense* being slightly irregularly linear-lanceolate, whereas the values of *A.pseudoconspicuum* are linear-elliptical and those of *A.initium* are linear-lanceolate to lanceolate. On the raphe valve, *A.anhuense* sp. nov. has a transapically rectangular or bow-tie central area, whereas the central area of *A.pseudoconspicuum* and *A.pyrenaicum* is small and hardly differentiated. *A.pseudoconspicuum* has narrow transverse fascia and *A.initium* has an asymmetrical transverse fascia central area. In addition, the axial area of *A.anhuense* sp. nov. is linear-lanceolate, but narrow linear in other similar species. The valves of *A.anhuense* sp. nov. are longer (13–35.7 µm) than in other similar species and wider (3.5–4.5 µm) than in *A.initium* (3.1–3.6 µm). Moreover, on the raphe valve, the striae density at the middle and apices of *A.anhuense* sp. nov. is less than in other similar species and, on the rapheless valve, the striae density at the middle of *A.anhuense* sp. nov. is less (16–26/10 µm) than in *A.initium* (32–35/10 µm). The striae density at the apices of *A.anhuense* sp. nov. is less (22–30/10 µm) than in *A.initium* (32–35/10 µm) and *A.pyrenaicum* (32–38/10 µm), but higher than in *A.pseudoconspicuum* (20–24/10 µm).

**Table 1.** Comparison of morphological characteristics of *Achnanthidiumanhuensis* sp. nov. and closely related taxa.

Species/Feature	*A.anhuensis* sp. nov.	*A.initium* Karthick, Taylor & Hamilton	*A.rostropyrenaicum* Jüttner & Cox	*A.pseudoconspicuum* (Foged) Jüttner & Cox	*A.pyrenaicum* (Hustedt) Kobayasi
Valve length (μm)	13–35.7	11–25.2	18–24.5	12.9–21	10–16
Valve width (μm)	3.5–4.5	3.1–3.6	4.3–4.5	2.9–4.8	2.5–4
Valve outline	Linear-lanceolate	Linear-lanceolate to lanceolate	Linear-lanceolate	Linear-elliptical	Linear-lanceolate
Valve apices	Rounded or weakly protracted	Rounded to weakly rostrate rounded	Rostrate	Rounded or slightly cuneate	Slightly drawn–out ends
**Raphe valve**					
Axial area	Narrow, linear-lanceolate	Narrow linear	Narrow linear	Narrow linear	Narrow linear
Central area	Rectangular or bow tie	Asymmet-rical transverse fascia	Small and hardly differentiated	Narrow transverse fascia	Small and hardly differentiated
Raphe	Distal fissures deflected to the same side, internally proximal raphe fissures distinct deflected in opposite direction	Distal fissures deflected to the opposite side at an angle of 80–90°, internally proximal ends curved in opposite directions	Raphe distal raphe ends curved to the same side, internally proximal raphe endings curved towards opposite side	Distal fissures deflected to the opposite side at an angle of 60–80°, internally central raphe ends curve to opposite sides	Raphe distal raphe ends curved to the same side, internally proximal raphe endings curved towards opposite side
Density of striae (10 μm)	18–20 (middle), 26–31 (apices)	29–34	20–22 (middle), 32 (apices)	22–24 (middle), 32 (apices)	20–25 (middle), 34–40 (apices)
Number of areolae per stria	3–6 (middle), 1–3 (apices)	2–5 (middle), 1–3 (apices)	2–6 (middle), 1–3 (apices)	3–5 (middle), 1–3 (apices)	No data
**Rapheless valve**					
Axial area	Narrow linear-lanceolate	Narrow linear	Narrow linear	Narrow linear	Narrow linear
Central area	Absent	Weakly elliptical to almost absent	Absent	Absent	Absent
Density of striae (10 μm)	16–26 (middle), 22–30 (apices)	32–35	22 (middle), 28 (apices)	20–24	20–28 (middle), 32–38 (apices)
Number of areolae per stria	4–6 (middle), 1–2 (apices)	3–5 (middle), 1–3 (apices)	4–6 (middle), 1–2 (apices)	4–6 (middle), 2–3 (apices)	No data
References	Current study	Karthick et al. (2017)	Jüttner et al. (2011)	Jüttner and Cox (2011)	Kobayashi (1997)

Species similar to *A.qingxiense* sp. nov. include *A.gracillimum* (Meister) Mayama (Kobayasi et al. 2006), *A.chitrakootense* Wojtal (Wojtal et al. 2010), *A.sinense* Liu & Blanco (Liu et al. 2016), *A.linannulumm* Karthick, Taylor & Hamilton (Karthick et al. 2017) and *A.sublanceolatum* Yu, You & Wang (Yu et al. 2019a). This group of species is compared in Table 2. Externally, on the raphe valve, *A.qingxiense* sp. nov. has a linear-lanceolate valve with rounded apices, while *A.linannulumm* possesses linear elliptical to lanceolate valves and rounded or slightly protracted apices. The species of *A.sinense* are not protracted, but are acute and round, *A.gracillimum* has an elliptical to lanceolate valve and narrowly rostrate to subcapitate apices and A.chitrakootense possesses linear to linear-elliptical valve and subcapitate to rounded apices. In addition, *A.qingxiense* sp. nov. has a narrow linear-lanceolate axial area, whereas the axial area of *A.gracillimum* is linear and that of *A.linannulumm* is lanceolate. *A.qingxiense* sp. nov. has absent central area, while *A.sinense* possesses a rhombic-lanceolate central area and *A.gracillimum* has an asymmetrical central area. Moreover, on the rapheless valve, *A.qingxiense* sp. nov. can easily be separated from other similar species, based on its being distinctly expanded at the apices on the axial area (Figs 8, 9). Conversely, on the raphe valve, the striae density at the middle of *A.qingxiense* sp. nov. is less (21–25/10 µm) than in *A.chitrakootense* (26–30/10 µm), but higher than in *A.gracillimum* (22/10 µm) and the striae density at the apices of *A.qingxiense* sp. nov. is higher than other similar species. On the rapheless valve, the striae density at the middle of *A.qingxiense* sp. nov. is less (20–24/10 µm) than in *A.linannulumm* (24–26/10 µm) and *A.chitrakootense* (26–30/10 µm), but higher than in *A.gracillimum* (22/10 µm) and the striae density at the apices of *A.qingxiense* sp. nov. is higher than other similar species.

**Table 2.** Comparison of morphological characteristics of *Achnanthidiumqingxiensis* sp. nov. and closely related taxa.

Species/Feature	*A.qingxiensis* sp. nov.	*A.sublanceolatum* Yu, You & Wang	*A.linannulumm* Karthick, Taylor & Hamilton	*A.sinense* Liu & Blanco	*A.gracillimum* (Meister) Mayama	*A.chitrakootense* Wojtal
Valve length (μm)	22.5–28	18–35	15.5–32.5	17.5–31.7	19–31.5	13–42
Valve width (μm)	3.8–4.6	4–4.5	2.5–4.5	4.1–6.0	3–4	3.4–4.2
Valve outline	Linear-lanceolate	Linear-lanceolate	Linear elliptical to lanceolate	Narrow lanceolate	Elliptical to lanceolate	Linear to linear-elliptical
Valve apices	Rounded	Rounded or weakly protracted	Rounded or slightly protacted	Not protracted, acute round	Narrowly rostrate to subcapitate	Subcapitate to rounded
**Raphe valve**						
Axial area	Narrow, linear-lanceolate	Narrow, linear-lanceolate	Lanceolate	Narrow lanceolate	Linear	linear-lanceolate
Central area	Absent	Absent	Indistinct to weakly expanded	Rhombic-lanceolate	Asymmetrical	Indistinct to weakly expanded
Raphe	Slightly undulate, distal raphe ends deflected towards the same side, internally proximal raphe endings weakly deflected in opposite directions	Distal raphe fissures deflected to the same side, internally proximal raphe fissures weakly deflected in opposite direction	Distal fissures deflected to the same side at an ange of 80–90°, internally proximal ends curved in opposite directions	Raphe distal raphe ends curved to the same side, internally proximal raphe endings curved towards opposite side	Distal raphe fissures are sharply bent, internally central raphe ends curve to opposite sides	Distal raphe ends strongly curved to the same side, proximal raphe ends with slightly defl ected to opposite sides
Density of striae (10 μm)	21–25 (middle), 42–44 (apices)	20–23 (middle), 34–42 (apices)	24–27 (middle), 32–34 (apices)	21–28 (middle), 40 (apices)	22 (middle), 36 (apices)	26–30
Number of areolae per stria	5–8 (middle), 1–3 (apices)	3–4 (middle), 1–2 (apices)	2–3	5–6 (middle), 1–3 (apices)	4–5 (middle), 1–2 (apices)	2–4 (middle), 1–3 (apices)
**Rapheless valve**						
Axial area	Linear	Narrow linear-lanceolate	Narrow lanceolate	Linear	Linear	Linear
Central area	Absent	Absent	Weakly expanded to absent	Absent	Weakly expanded to absent	Absent
Density of striae (10 μm)	20–24 (middle), 32–34 (apices)	21–24 (middle), 30–36 (apices)	24–26 (middle), 28–30 (apices)	21–27 (middle), 34 (apices)	22 (middle), 36 (apices)	26–30
Number of areolae per stria	5–10 (middle), 1–4 (apices)	2–5 (middle), 1–2 (apices)	2–4	6–7 (middle), 1–3 (apices)	4–5 (middle), 1–3 (apices)	4–5 (middle), 2–4 (apices)
References	Current study	Yu et al. (2019a)	Karthick et al. (2017)	Liu et al. (2016)	Kobayasi et al. (2006)	Wojtal et al. (2010)

*A.anhuense* sp. nov. and *A.qingxiense* sp. nov. were collected from stones in Qingxi River, which is fast-flowing. *Achnanthidium* species can occur across a broad range of trophic conditions, from oligotrophic to eutrophic waters (Karthick et al. 2017; Miao et al. 2020). The *A.pyrenaicum* complex is abundant in clear and fast- flowing streams and they can be good indicators of specific environmental conditions in freshwater ecosystems (Cantonati and Spitale 2009; Jüttner et al. 2011), which is supported by the results of this study. The two new species occur in low TN (0.5 mg·l^-1^), TP (0.03 mg·l^-1^) and COD (0.1 mg·l^-1^) environments. Moreover, in the type locality, some other monoraphid species co-occur with these new species. The co-occurring monoraphid taxa include *A.latecephalum* Kobayasi (Kobayasi 1997), *A.pyrenaicum* (Hustedt) Kobayasi (Kobayasi 1997), *Planothidiumlanceolatum* (Brébisson ex Kützing) Lange-Bertalot (Lange-Bertalot 1999), *A.rivulare* Potapova & Ponader (Potapova and Ponader 2004) and A.subhudsonisvar.kraeuselii (Cholnoky) Cantonati & Lange-Bertalot (Kusber et al. 2017). In further studies, we will continue to study the relationship between diatom diversity and ecology from this region.

## Supplementary Material

XML Treatment for Achnanthidium anhuense

XML Treatment for Achnanthidium qingxiense

## References

[B1] Bory de Saint-Vincent JBGN (1822) Achnanthe. *Achnanthes.* Dictionnaire Classique d’Histoire Naturelle 1: 79–80.

[B2] Cantonati M Spitale D (2009) The role of environmental variables in structuring epiphytic and epilithic diatom assemblages in spring and streams of the Dolomiti Bellunesi National Park (south-eastern Alps). Fundamental and Applied Limnology 174(2): 117–133. 10.1127/1863-9135/2009/0174-0117

[B3] CSEPB [Chinese State Environment Protection Bureau] (2002) Water and wastewater monitoring analysis methods, 4^nd^ edn. Chinese Environment Science Press, Beijing, China.

[B4] Compère P Van de Vijver B (2011) *Achnanthidiumennediense* (Compère) Compère et Van de Vijver comb. nov. (Bacillariophyceae), the true identity of *Naviculaennediensis* compère from the Ennedi Mountains (Republic of Chad). Algological Studies 136/137: 5–17. 10.1127/1864-1318/2011/0136-0005

[B5] Hustedt F (1922) Bacillariales aus Innerasien. Gesammelt von Dr. Sven Hedin. In: Hedin S (Ed.) Southern Tibet, discoveries in former times compared with my own researches in 1906–1908.Lithographic Institute of the General Staff of the Swedish Army, Stockholm 6(3), 107–152. 10.5962/bhl.title.64226

[B6] Jao C (1964) Some fresh-water algae from southern Tibet. Oceanologia et Limnologia Sinica 6(2): 169–192.

[B7] Jao C Zhu H Lee Y (1974) The fresh-water algae from Mount Qomolangma District (in Tibet). Report of the Scientific Survey of Mount Qomolangma District 1966–1968: 92–126.

[B8] Jüttner I Cox EJ (2011) *Achnanthidiumpseudoconspicum* comb. nov.: Morphology and ecology of the species and a comparison with related taxa. Diatom Research 26(1): 21–28. 10.1080/0269249X.2011.573707

[B9] Jüttner I Chimonides J Cox J (2011) Morphology, ecology and biogeography of diatom species related to *Achnanthidiumpyrenaicum* (Hustedt) Kobayasi (Bacillariophyceae) in streams of the Indian and Nepalese Himalaya. Algological Studies 136/137: 45–76. 10.1127/1864-1318/2011/0136-0045

[B10] Karthick B Taylor JC Hamilton PB (2017) Two new species of *Achnanthidium* Kützing (Bacillariophyceae) from Kolli Hills, Eastern Ghats, India. Fottea 17(1): 65–77. 10.5507/fot.2016.020

[B11] Kobayasi H (1997) Comparative studies among four linear-lanceolate *Achnanthidium* species (Bacillariophyceae) with curved terminal raphe endings. Nova Hedwigia 65(1–4): 147–164. 10.1127/nova.hedwigia/65/1997/147

[B12] Kobayasi H Idei M Mayama S Nagumo T Osada K (2006) H. Kobayasi’s Atlas of Japanese Diatoms based on electron microscopy. Uchida Rokakuho Publishing Co., Ltd, Tokyo, 531 pp.

[B13] Kociolek JP Balasubramanian K Blanco S Coste M Ector L Liu Y Kulikovskiy M Lundholm N Ludwig T Potapova M Rimet F Sabbe K Sala S Sar E Taylor J Van de Vijver B Wetzel CE Williams DM Witkowski A Witkowski J (2018) In DiatomBase. http://www.diatombase.org [accessed on 2018-03-15]

[B14] Kulikovskiy MS Maltsev YI Glushchenko AM Kuznetsova IV Kapustin DA Lange-Bertalot H Genkal SI Kociolek JP (2020) *Gogorevia*, a new monoraphid diatom genus for *Achnanthesexigua* and allied taxa (Achnanthidiaceae) described on the basis of an integrated molecular and morphological approach. Journal of Phycology 56(6): 1601–1613. 10.1111/jpy.13064 32871027

[B15] Kusber WH Cantonati M Lange-Bertalot H (2017) Validation of five diatom novelties published in “Freshwater Benthic Diatoms of Central Europe” and taxonomic treatment of the neglected species *Tryblionellahantzschiana*. Phytotaxa 328(1): 90–94. 10.11646/phytotaxa.328.1.6

[B16] Kützing FT (1844) Die Kieselschaligen Bacillarien oder Diatomeen. W. Köhne, Nordhausen, 152 pp. 10.5962/bhl.title.64360

[B17] Lange-Bertalot H (1999) Neue Kombinationen von Taxa aus *Achnanthes* Bory (sensu lato). Iconographia Diatomologica 6: 270–283.

[B18] Liu B Blanco S Long H Jingjing XU Jiang X (2016) *Achnanthidiumsinense* sp. nov. (Bacillariophyta) from the Wuling Mountains Area, China. Phytotaxa 284(3): 194–202. 10.11646/phytotaxa.284.3.4

[B19] Liu Y Tan X Kociolek JP Kulikovskiy M Lu XX Fan YW (2021) One new species of *Achnanthidium* Kützing (Bacillariophyta, Achnanthidiaceae) from the upper Han River, China. Phytotaxa 516(2): 187–194. 10.11646/phytotaxa.516.2.6

[B20] Marquardt GC Costa LF Bicudo DC Bicudo CEDM Blanco S Wetzel CE Ector L (2017) Type analysis of *Achnanthidiumminutissimum* and *A.catenatum* and description of *A.tropicocatenatum* sp. nov. (Bacillariophyta), a common species in Brazilian reservoirs. Plant Ecology and Evolution 150(3): 313–330. 10.5091/plecevo.2017.1325

[B21] Miao M Li Z Hwang EA Kim HK Lee H Kim BH (2020) Two new benthic diatoms of the genus *Achnanthidium* (Bacillariophyceae) from the Hangang River, Korea. Diversity (Basel) 12(7): e285. 10.3390/d12070285

[B22] Novais MH Hlúbiková D Morais M Hoffmann L Ector L (2011) Morphology and ecology of *Achnanthidiumcaravelense* (Bacillariophyceae), a new species from Portuguese rivers. Algological Studies 136: 131–150. 10.1127/1864-1318/2011/0136-0131

[B23] Parr JF Taffs KH Lane CM (2004) A microwave digestion technique for the extraction of fossil diatoms from coastal lake and swamp sediments. Journal of Paleolimnology 31(3): 383–390. 10.1023/B:JOPL.0000021857.32734.c6

[B24] Pérès F Cohu RL Delmont D (2014) *Achnanthidiumbarbei* sp. nov. and *Achnanthidiumcostei* sp. nov., two new diatom species from French rivers. Diatom Research 29(4): 387–397. 10.1080/0269249X.2014.890956

[B25] Pinseel E Van de Vijver B Kopalova K (2015) *Achnanthidiumpetuniabuktianum* sp. nov. (Achnanthidiaceae, Bacillariophyta), a new representative of the *A.pyrenaicum* group from Spitsbergen (Svalbard Archipelago, High Arctic). Phytotaxa 226(1): 63–74. 10.11646/phytotaxa.226.1.6

[B26] Potapova MG Ponader KC (2004) Two common North American diatoms, *Achnanthidiumrivulare* sp. nov. and *A.deflexum* (Reimer) Kingston: Morphology, ecology and comparison with related species. Diatom Research 19(1): 33–57. 10.1080/0269249X.2004.9705606

[B27] Qi YZ Xie SQ (1984) The diatom in moss swamp from Hubei Shennongjia. Jinan Yili Xuebao 1984(3): 86–92.

[B28] Round FE Bukhtiyarova L (1996) Four new genera based on Achnanthes (Achnanthidium) together with re-definition of *Achnanthidium.* Diatom Research 11(2): 345–361. 10.1080/0269249X.1996.9705389

[B29] Round FE Crawford RM Mann DG (1990) The Diatoms. Biology and morphology of the genera. Cambridge University Press, Cambridge, 747 pp.

[B30] Tseplik ND Maltsev YI Glushchenko AM Kuznetsova IV Genkal SI Kociolek JP Kulikovskiy MS (2021) *Achnanthidiumtinea* sp. nov.-a new monoraphid diatom (Bacillariophyceae) species, described on the basis of molecular and morphological approaches. PhytoKeys 174: 147–163. 10.3897/phytokeys.174.60337 33776528 PMC7979678

[B31] Wojtal AZ Lange-Bertalot H Nautiyal R Verma J Nautiyal P (2010) *Achnanthidiumchitrakootense* spec. nov. from rivers of northern and central India. Polish Botanical Journal 55: 55–64.

[B32] You QM Cao Y Yu P Kociolek JP Zhang LX Wu B Lowe R Wang QX (2019) Three new subaerial *Achnanthidium* (Bacillariophyta) species from a karst landform in the Guizhou Province, China. Fottea 19(2): 138–150. 10.5507/fot.2019.005

[B33] You QM Zhao K Wang YL Yu P Kociolek JP Pang WT Wang QX (2021) Four new species of monoraphid diatoms from Western Sichuan Plateau in China. Phytotaxa 479(3): 257–274. 10.11646/phytotaxa.479.3.3

[B34] Yu P You QM Kociolek JP Lowe R Wang QX (2017) *Nupelamajor* sp. nov. a new diatom species from Maolan Nature Reserve, central-south of China. Phytotaxa 311(3): 245–254. 10.11646/phytotaxa.311.3.4

[B35] Yu P Kociolek JP You QM Wang QX (2018) *Achnanthidiumlongissima* sp. nov. (Bacillariophyta), a new diatom species from Jiuzhai Valley, southwestern China. Diatom Research 33(3): 339–348. 10.1080/0269249X.2018.1545704

[B36] Yu P You Q Kociolek JP Wang Q (2019a) Three new freshwater species of the genus *Achnanthidium* (Bacillariophyta, Achnanthidiaceae) from Taiping Lake, China. Fottea 19(1): 33–49. 10.5507/fot.2018.015

[B37] Yu P You QM Pang WT Cao Y Wang QX (2019b) Five new Achnanthidiaceae species (Bacillariophyta) from Jiuzhai Valley, Sichuan Province, southwestern China. Phytotaxa 405(3): 147–170. 10.11646/phytotaxa.405.3.5

[B38] Zhu HZ Chen JY (1994) Study on the diatoms of the Wuling Mountain Region. Compilation of reports on the survey of algal resources, 405 pp.

[B39] Zhu HZ Chen JY (1996) New taxa of diatom (Bacillariophyta) from Xizang (Tibet). (II). Zhiwu Fenlei Xuebao 34(1): 102–104.

